# The first COVID-19 new graduate nurses generation: findings from an Italian cross-sectional study

**DOI:** 10.1186/s12912-022-00885-3

**Published:** 2022-05-03

**Authors:** Alvisa Palese, Anna Brugnolli, Illarj Achil, Elisa Mattiussi, Stefano Fabris, Satu Kajander-Unkuri, Valerio Dimonte, Luca Grassetti, Matteo Danielis

**Affiliations:** 1 Department of Medical Sciences, Udine University, Viale Ungheria 20, 33100 Udine, Italy; 2 Department of Public Health, Azienda Provinciale per i Servizi Sanitari, Trento, Italy; 3 Department of Nursing Science, University of Turku, Turku, Finland; 4 Diaconia University of Applied Sciences, Helsinki, Finland; 5 Department of Public Health and Pediatrics, University of Turin, Turin, Italy

**Keywords:** COVID-19, Nursing education, Nursing students, New graduates, Cross-sectional study, Competences

## Abstract

**Background:**

Nursing education has been disrupted by the onset of the COronaVIrus Disease 19 (COVID-19) pandemic, potentially impacting learning experiences and perceived competencies at the time of graduation. However, the learning experiences of students since the onset of COVID-19, their perceived competences achieved and the employment status one month after graduation, have not been traced to date.

**Methods:**

A cross sectional online survey measured the individual profile, the learning experience in the last academic year and the perceived competences of the first COVID-19 new nursing graduates in two Italian universities. Details relating to employment status and place of employment (Covid-19 versus non-COVID-19 units) one month after graduation were also collected and the data compared with those reported by a similar cohort of new graduates pre-pandemic in 2018–2019. All those who graduated in November 2020 and attended their third year after the onset of the COVID-19 pandemic were eligible. The online survey included individual, nursing programme and first working experience variables alongside the Nurse Competence Scale (NCS). Descriptive and inferential statistical analyses were performed.

**Results:**

A total of 323 new graduates participated. In their last academic year, they experienced a single, long clinical placement in non-COVID-19 units. One month after graduation, 54.5% (*n* = 176) were working in COVID-19 units, 22.9% (*n* = 74) in non-COVID-19 units and 22.6 (*n* = 73) were unemployed. There was no statistical difference among groups regarding individual variables and the competences perceived. Fewer new graduates working in COVID-19 units experienced a transition programme compared to those working in non-COVID-19 units (*p* = 0.053). At the NCS, the first COVID-19 new graduate generation perceived significantly lower competences than the pre-COVID-19 generation in the ‘Helping role’ factor and a significant higher in ‘Ensuring quality’ and ‘Therapeutic interventions’ factors.

**Conclusions:**

The majority of the first COVID-19 new graduate generation had been employed in COVID-19 units without clinical experience and transition programmes, imposing an ethical debate regarding (a) the role of education in graduating nurses in challenging times with limited clinical placements; and (b) that of nurse managers and directors in ensuring safe transitions for new graduates. Despite the profound clinical placement revision, the first COVID-19 new graduate generation reported competences similar to those of the pre-COVID-19 generation, suggesting that the pandemic may have helped them to optimise the clinical learning process.

**Supplementary Information:**

The online version contains supplementary material available at 10.1186/s12912-022-00885-3.

## Background

From the onset of the COronaVIrus Disease 19 (COVID-19) pandemic in March 2020 [1], nursing programmes in Italy have been exposed to a tremendous stress-test and forced modifications to their well-established educational processes. Since 1994, Italian undergraduate nursing programmes consist of taught modules within the university and clinical placements within the National Health Services (NHS) [2]. Specifically, the third and final academic year has been designed to prepare students to identify nursing care needs and prioritise interventions among critically ill patients and those cared for in the community (mental health included) and paediatric settings, as well as to ensure patient safety and evidence-based care [3]. After the theoretical education, around > 550 h of clinical practice in critical care, mental health, paediatric, general medical, surgical and community care settings are offered according to European Directives [4], harmonising education across Europe. By law, graduation requirements have also been established as homogenous across universities, both in methods (examination regarding the competences expected and thesis dissertation) and time (November, first session; April, second session).

With the onset of the first COVID-19 wave [1], nursing education in the classroom was interrupted and transformed into online synchronous teaching. On the side of clinical learning, despite several national laws [5] recommending that clinical placements would have to be continued, Health Care Trusts imposed the interruption of clinical rotations to all health care students, mainly due to the lack of personal protective equipment and to prevent units’ overcrowding [6]. As a consequence, more than 50,000 nursing students were left at home; initiatives were suddenly implemented by nursing faculties and clinical training were re-established in a number of regions, while not in others [7]. Clinical placement priority was given to third year students because they were better equipped and also to enable them to graduate sooner to address the dramatic shortage in the NHS. However, as a consequence of the health service transformation, several units devoted to nursing education were disrupted: the number of available clinical placements was reduced, and the majority of health care facilities denied students access to COVID-19 areas. To minimise cross contamination the universities introduced a series of changes to clinical placements. These placements were limited to one or two long experiences (> 8 weeks) in non-Covid-19 areas with a small number of students in each area[5]. By law [8], universities have been allowed to compensate for the lack of opportunities in real-world settings by offering distance learning for clinical modules. No more than 40% of the time devoted to clinical rotations were allowed to be delivered online, and small groups of students to promote their active participation (e.g. debating clinical scenarios) were involved. At graduation, the first COVID-19 new graduate generation was recruited immediately to face the pandemic.

The changes undertaken in the patterns of nursing education on a large scale, and in that of the first employment experience, are unprecedented. Nursing education has been re-designed mainly around student safety and infectious disease control principles; by implementing urgent responses mixing online and limited clinical exposure in contexts undergoing dramatic changes in their mission (e.g. changing from medical to COVID-19 patients), in staffing (e.g. high turn-over) and in the patients cared for, given that several surgical procedures and other programmed activities were suspended or delayed [9, 10]. Moreover, no assessments have been performed on the quality of clinical environments to maximise the clinical competences achieved [11, 12]; also, new clinical tutors have been appointed without any training [13], and the continuity of the clinical experience has not always been ensured, due to the continuous changes in the mission of units and episodes of isolation or quarantine [14]. Furthermore, only limited transition programmes have been offered [15] to help new graduates to enter their first working environments using supervision and peer support in good clinical settings [16].

The main features of the clinical experiences attended by post-COVID-19 third-year students and their employment status and placement one month after graduation have been not documented to date; similarly, no data on perceived competences possessed by the first COVID-19 new graduate generation have been traced to date. Specifically, some individual variables, or learning experiences during the COVID-19 pandemic, and competences perceived are assumed to differ according to the employment status and placement of new graduates one month after graduation: for example, being employed in a COVID-19 unit may have decreased the perception of competences, given the absence of learning opportunities in these settings as compared to those working in non-COVID-19 units or still unemployed. Therefore, we undertook an exploratory study investigating the last year of clinical education up to graduation and one month after among the first COVID-19 new graduate generation. Specifically, the primary aim was to detect any differences at the individual and nursing programme levels and between the competences perceived among new graduates according to their working status (in COVID-19 units, in non-COVID-19 units and unemployed) one month after graduation. The secondary aim was to compare the perceived competences of the first COVID-19 new graduate generation with those reported by the pre-COVID-19 generation.

## Methods

### Study design

A cross-sectional on-line survey (December 2020–January 2021) was performed according to the Strengthening the Reporting of Observational Studies in Epidemiology checklist [17].

### Setting and sample

Two universities located in the North of Italy, where the majority of cases and deaths have been reported both in the first and in the second wave of the COVID-19 outbreak [18, 19], already collaborating in a research network (e.g., [20]) to measure the competence perceived by new graduates [21], were approached. Nursing programmes were homogenous in duration (three years) and in the main curriculum contents [2]. In each programme, those students who had just graduated in November 2020 were considered eligible if they were (a) attending the third and final year at the onset of the COVID-19 pandemic and (b) willing to participate in the survey. Therefore, students delaying their graduation, as well as those who were not willing to participate, were not involved. Eligible new graduates (*n* = 631) were approached via email, and the inclusion of participants was stopped one month after graduation to prevent any recall bias and when > 300 new graduates had agreed to participate, mirroring the sample size of a previous survey performed in the same nursing programmes on competences perceived by new graduates [21].

### Questionnaire

The questionnaire (Supplementary Table 1) was composed of different sections, assessing variables at the following levels:

1. individual (e.g. age, gender), 10 questions;
2. employment status and place of employment one month after graduation (working in non-COVID-19 units, working in COVID-19 units or unemployed) and the number of working offers received, one question;
3. nursing programme (e.g. duration of clinical rotations from the COVID-19 onset to graduation), nine questions;
4. first working experience as a new graduate (e.g. transition programme received or not, number of patients cared for, discharged or who died in the last shift before filling in the survey), four questions, and
5. competences perceived as measured with the Nurse Competence Scale (NCS) documented to be capable of measuring the generic competences of nurses as the functional adequacy and capacity to integrate knowledge, skills, attitudes and values in specific contextual situations [22] (Supplementary Table 1). The NCS is composed of two sections: (a) the first composed of 73 items (eight factors) for which the nurse is asked to rate the level of competence perceived by using a visual analogue scale (VAS 0‒100; 0 = low level, 100 = high level of competence) and (b) the second section, were nurses are asked to rank the frequency of use of each competence in their clinical training (from ‘Not applicable to ‘Used very often in my work’). In Supplementary Table 1, a full description of the tool, also with reliability and validity data, is summarised.

In order to explore differences, if any, in the competences perceived by the first COVID-19 new graduate generation compared with the pre-COVID-19 generation, all data collected were compared to those collected in 2018–2019 in the programmes briefly summarised in Supplementary Table 2 [21].

The questionnaire composed of multiple sections was developed according to the available literature on nursing education in the time of COVID-19 [23, 24], a previous survey in the field [21] and the experience of the research team. The questionnaire was firstly reviewed by the research team (see authors), and then it was piloted among non-eligible new graduated nurses in order to assess its clarity and feasibility; no changes were suggested.

### Data collection

The online web questionnaire was launched by a researcher appointed to each university, two weeks after graduation (e.g. in Udine University, on 14 December 2020) and left open for one month (up to 14 January 2021). Three email reminders were sent to all eligible new graduates.

### Data analysis

Descriptive and inferential statistics were performed by calculating frequencies, percentages and averages (Confidence Interval [CI] 95%). Data were stratified and compared in the following sub-groups of nurses: (a) working in COVID-19 units; (b) working in non-COVID-19 units and (c) unemployed. Differences, if any, across all groups and between pairs of groups (employed vs*.* unemployed) were explored according to the nature of the variables: (a) in the case of dichotomous variables, the chi-square test (or Fisher’s exact test, when appropriate) was used, and (b) in the case of continuous variables, their distribution was visually explored for normality in a preliminarily fashion, and then ANOVA was used to test for overall differences across groups. Then, post hoc tests between two groups were performed in cases where statistical significances emerged in the ANOVA test. To ensure a parsimonious approach, the results of post hoc testing have been reported in the text and in the tables only in the case of statistical significance. Moreover, in order to assess differences, if any, in the perception of competences of the first COVID-19 new graduate generation compared with that pre-COVID-19, a comparison was made by using the t-test with data collected from the same programmes [21] after having checked participant homogeneity (e.g. age). All analyses were performed with SPSS Package, version 27 (IBM Corp., Armonk, NY) and with R core software (R Core Team, 2021) [25].

### Ethical issues

The questionnaire was sent via a university reference researcher to all eligible new graduates. The participation was voluntary, and no rewards were offered. In its introduction section, the aims of the study, the confidentiality of the data collected and anonymity with regard to each participant and the health care facilities where they were employed, were all ensured. Each participant was invited to formally express his/her consent to participate before accessing the questions; then, the questionnaire was displayed, allowing participants to complete it.

## Results

### Participants’ characteristics, employment status and location

**Fig. 1:**
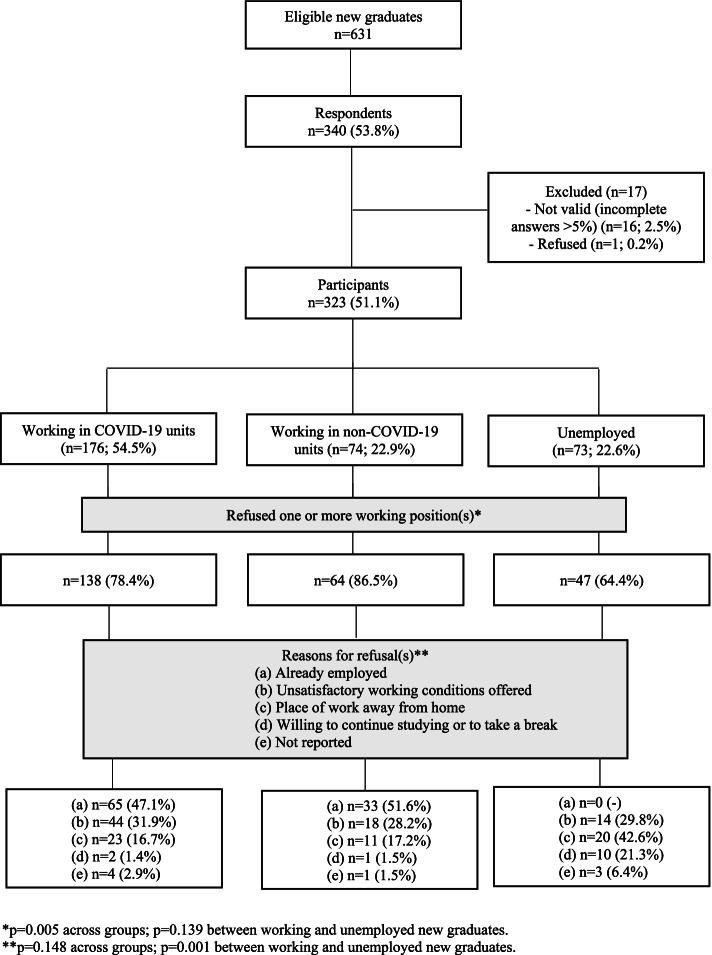
Flow diagram of participants according to their working profile

A total of 323 new graduates participated; only one refused. One month after graduation, 176 (54.5%) were working in COVID-19 units, 74 (22.9%) in non-COVID-19 units and 73 (22.6%) were unemployed (Fig. 1). Most participants (*n* = 249; 77.1%) refused one or more job offers; this was true significantly more often for nurses working in non-COVID-19 settings (*n* = 64; 86.5%) than for those who were working in COVID-19 units (*n* = 138; 78.4%) and unemployed nurses (*n* = 47; 64.4%) (*p* = 0.005), while no differences emerged between employed and non-employed nurses (*p* = 0.139). Reasons for refusal were homogeneous across groups (*p* = 0.148) but significantly different between working new graduates (namely, already employed, and unsatisfactory working conditions offered) and those unemployed (distance from home) (*p* = 0.001).

### Individual variables according to the employment status and location

Participants were, on average, 23.51 years old (CI 95% 23.18–23.84); the majority were female (*n* = 287; 88.9%) and living with their families (*n* = 254; 78.6%). Moreover, the majority reported neither previous university (*n* = 253; 78.3%) nor work experience(s) (*n* = 135; 41.8%). At the time of onset of the COVID-19 pandemic, they were attending lessons (*n* = 140; 43.3%) or clinical training (*n* = 112; 34.7%); moreover, the number of clinical experiences attended up to the COVID-19 outbreak was, on average, 5.15 (CI 95% 5.00–5.29). No statistical differences between groups emerged according to employment status and place of employment for individual variables, as reported in Table 1.

**Table 1:** Participants, since the COVID-19 outbreak according to the employment status and location (*n* = 323)

Individual variables	Overall (*n* = 323)	Working in COVID-19 units (*n* = 176)	Working in non-COVID-19 units (*n* = 74)	Unemployed (*n* = 73)	COVID-19 vs. non COVID-19 units vs. unemployed *p-value*^*a*^	Post-hoc testing *p-value*^*ab*^
Age (years), mean (CI95%)	23.51 (23.18–23.84)	23.41 (23.04–23.78)	23.58 (23.09–24.07)	23.67 (22.60–24.74)	0.801	-
Gender, female, n (%)	287 (88.9)	152 (86.4)	65 (87.8)	70 (95.9)	0.089	-
Living with, n (%)						
With my family	254 (78.6)	135 (76.7)	58 (78.4)	61 (83.6)	0.506	-
With my boyfriend/girlfriend	42 (13.0)	22 (12.5)	11 (14.9)	9 (12.3)		
Alone	13 (4.0)	10 (5.7)	1 (1.3)	2 (2.7)		
Students/colleagues	14 (4.4)	9 (5.2)	4 (5.4)	1 (1.4)		
With Children, n (%)	6 (1.9)	3 (1.7)	1 (1.4)	2 (2.7)	0.803	-
Secondary education, n (%)						
High school	245 (75.9)	131 (74.4)	54 (73.0)	60 (82.2)	0.554	-
Technical School	52 (16.1)	32 (18.2)	12 (16.2)	8 (10.9)		
Professional School	24 (7.4)	12 (6.8)	8 (10.8)	4 (5.5)		
Foreign School	2 (0.6)	1 (0.6)	0	1 (1.4)		
Secondary school, n (%)						
Grade (score 60–100), mean (CI95%)	79.27 (78.26–80.29)	79.52 (78.11–80.92)	77.97 (75.78–80.17)	80.01 (78.03–82.00)	0.360	-
Previous universities experience, n (%)						
None	253 (78.3)	135 (76.7)	60 (81.1)	58 (79.5)	0.598	-
Bachelor in other fields concluded	21 (6.5)	15 (8.5)	4 (5.4)	2 (2.7)		
Bachelor in other fields interrupted	43 (13.3)	24 (13.7)	8 (10.8)	11 (15.1)		
Other	6 (1.8)	2 (1.1)	2 (2.7)	2 (2.7)		
Previous work experience, n (%)	135 (41.8)	67 (38.1)	31 (41.9)	37 (50.7)	0.185	-
Academic activities followed at the COVID-19 outbreak onset, n (%)						
I was following lesson	140 (43.3)	69 (39.2)	28 (37.8)	43 (58.9)	0.089	-
I was attending clinical placements	112 (34.7)	67 (38.0)	25 (33.8)	20 (27.4)		
I was attending examination(s)	59 (18.3)	33 (18.8)	18 (24.3)	8 (11.0)		
I was abroad (Erasmus experience)	9 (2.8)	4 (2.3)	3 (4.1)	2 (2.7)		
Other	3 (0.9)	3 (1.7)	0	0		
Clinical placements attended, number, mean (CI95%) up to the COVID-19 outbreak onset	5.15 (5.00–5.29)	5.04 (4.83–5.25)	5.15 (4.82–5.49)	5.40 (5.16–5.63)	0.152	-

*CI* Confidence Interval, *COVID-19* COronaVIrus Disease

^a^ANOVA Test for continuous variables, Chi Square (Fisher when appropriate) for dichotomous variables

^b^Post hoc testing (according to the nature of the variables)

### Nursing programme variables according to the employment status and location

As reported in Table 2, after the COVID-19 outbreak, students attended around 9.44 (CI 95% 8.79–10.10) weeks in the units homogenously across groups (*p* = 0.130), while an average of 4.47 weeks (CI 95% 3.26–7.68) were spent on distance learning, and there was no difference across groups (*p* = 0.122). Most students were not exposed to COVID-19 patients before their graduation (*n* = 293; 90.7%), and there was no statistical difference across groups differing in employment status and location (*p* = 0.846). Moreover, some areas (e.g. operating rooms) were also restricted in their access (*n* = 189; 58.5%), with no difference across groups (*p* = 0.146).

**Table 2:** Participants and the COVID-19 outbreak: clinical experiences from the COVID-19 onset up to graduation, according to their employment status and location (*n* = 323)

Clinical placements from COVID-19 onset (March 2020) to graduation (December 2020)	Overall (*n* = 323)	Working in COVID-19 units (*n* = 176)	Working in non-COVID-19 units (*n* = 74)	Unemployed (*n* = 73)	COVID-19 vs. non COVID-19 units vs. unemployed *p-value*^*a*^	Post-hoc testing *p-value* ^a,b^
Learning experiences attended						
At the ward level, weeks	9.44 (8.79–10.10)	8.88 (8.04–9.71)	9.74 (8.14–11.34)	10.51 (9.17–11.84)	0.130	-
On distance, weeks	4.47 (3.26–7.68)	4.52 (3.66–5.37)	4.03 (2.70–5.35)	2.90 (1.68–4.13)	0.122	-
Attended units/hospitals, n (%)						
Never caring for COVID + patients	293 (90.7)	161 (91.5)	66 (89.2)	66 (90.4)	0.846	-
Caring for COVID + patients	30 (9.3)	15 (8.5)	8 (10.8)	7 (9.6)		
Not allowed to access COVID + units	189 (58.5)	108 (61.4)	36 (48.6)	45 (61.6)	0.146	-
Preceptorship model, n (%) I was supervised by						
A clinical nurse	229 (70.9)	124 (70.5)	55 (74.3)	50 (68.5)	0.738	-
The nursing staff	62 (19.2)	32 (18.2)	14 (18.9)	16 (21.9)		
Nurse identified daily	6 (1.9)	5 (2.8)	0	1 (1.4)		
The nurse teacher	5 (1.5)	4 (2.3)	1 (1.4)	0		
The head nurse	21 (6.5)	11 (6.3)	4 (5.4)	6 (8.2)		
Perceived safety, n (%)						
Not at All	3 (0.9)	3 (1.7)	0	0	0.443	-
Very Little	21 (6.5)	11 (6.3)	7 (9.5)	3 (4.1)		
Somewhat	172 (53.3)	90 (51.1)	43 (58.1)	39 (53.4)		
To a Great Extent	127 (39.3)	72 (40.9)	24 (32.4)	31 (42.5)		
Perceived preparedness to deal with the clinical rotation, n (%)						
Not at All	11 (3.4)	7 (4.0)	3 (4.0)	1 (1.4)	0.198	-
Very Little	55 (17.0)	34 (19.3)	15 (20.3)	6 (8.2)		
Somewhat	174 (53.9)	88 (50.0)	42 (56.8)	44 (60.3)		
To a Great Extent	83 (25.7)	47 (26.7)	14 (18.9)	22 (30.1)		
Interruptions for quarantine						
Yes, for COVID + cases among patients’/health care workers, n (%)	11 (3.4)	8 (4.5)	2 (2.7)	1 (1.4)	0.006	-
Yes, for COVID + cases among out of hospital contacts, n (%)	10 (3.1)	2 (1.1)	7 (9.5)	1 (1.4)		
Clinical training interruptions, number, mean (CI95%)	1.07 (0.97–1.17)	1.07 (0.92–1.21)	1.11 (0.85–1.37)	1 (1.00–1.00)	0.240	-
Contagion, n (%)						
Yes, during my clinical placements	1 (0.3)	0	1 (1.4)	0	0.028	0.005^d^
Yes, at home	14 (4.3)	3 (1.7)	7 (9.5)	4 (5.5)		
I don’t know (I was not tested)	40 (12.4)	24 (13.6)	11 (14.8)	5 (6.8)		
No, never	268 (83.0)	149 (84.7)	55 (74.3)	64 (87.7)		
Nursing Programme degree of satisfaction regarding the COVID-19 outbreak management						
Not at All	11 (3.4)	3 (1.7)	6 (8.1)	2 (2.7)	0.043	0.816^d^
Very Little	76 (23.5)	44 (25.0)	18 (24.3)	14 (19.2)		
Somewhat	198 (68.3)	103 (58.5)	42 (56.8)	53 (72.6)		
To a Great Extent	38 (11.8)	26 (14.8)	8 (10.8)	4 (5.5)		
Final grade^c^, mean (CI95%)	103.4 (102.6–104.2)	104.0 (103.0–105.0)	102.2 (100.5–103.9)	103.1 (101.4–104.7)	0.143	-

*CI* Confidence Interval, *COVID-19* COronaVIrus Disease

^a^ANOVA Test for continuous variables, Chi Square (Fisher when appropriate) for dichotomous variables

^b^Post hoc-testing (tests according to the nature of the variables)

^c^Final grade obtained at graduation: from 60 (minimum) to 110 (maximum) cum laude

^d^Employed vs. unemployed newly graduates

During their clinical learning experiences, students were supervised by clinical nurses (*n* = 229; 70.9%) homogenously across groups (*p* = 0.738). In attending the clinical placements, they perceived themselves as from ‘Somewhat safe’ to ‘Very safe’ (*n* = 299; 92.6%), which was homogeneous across groups differing in employment status and location (*p* = 0.443); moreover, they also perceived themselves as prepared (from ‘Somewhat’ to ‘A great extent’, *n* = 257; 79.6%), and in this case, there were also no statistical differences across groups (*p* = 0.198).

However, clinical rotations were interrupted due to student quarantine more often among new graduates working in COVID-19 units (*n* = 8; 4.5%) than in those working in non-COVID-19 units (*n* = 2; 2.7%) and unemployed (*n* = 1; 1.4) (*p* = 0.006). Clinical rotation interruptions were, on average, 1.07 (CI 95% 0.97–1.17) and homogeneous across groups (*p* = 0.240). Furthermore, 15 participants (4.6%) reported COVID-19 test positivity, nearly all due to family reasons (*n* = 14; 4.3%). Test positivity was reported significantly more often (*p* = 0.028,) among those working in non-COVID-19 units (*n* = 7; 9.5%) than in those employed in COVID-19 units (*n* = 3; 1.7%) or unemployed (*n* = 4; 5.5%), (*p* = 0.005). Employed new graduates were significantly more likely to have had a positive COVID-19 test often due to family reasons, as compared to those who were unemployed (*p* = 0.005).

The degree of satisfaction with the outbreak management provided by the nursing programme was significantly higher among unemployed nurses (from ‘Somewhat’ to a ‘Great extent’, *n* = 57;78.1%) compared to those working in COVID-19 (*n* = 129; 73.3%) and non-COVID-19 units (*n* = 50; 67.2%) (*p* = 0.043). However, no significant differences emerged in the degree of satisfaction among employed and unemployed graduates (*p* = 0.816). The final grade at graduation was homogeneous across groups (*p* = 0.143).

### Working experience at one month after graduation

As reported in Table 3, fewer nurses working in COVID-19 units had experienced a transition programme compared to those working in non-COVID-19 units (*n* = 145; 82.5% vs. 68; 91.9%; *p* = 0.053), and the duration of the transition programme was around 18/19 shifts, with no statistical difference between groups (*p* = 0.783). Around 31.2% of those working in COVID-19 and 29.8% of those working in non-COVID-19 units perceived themselves as not ready at all or insufficiently ready to take on the responsibility of patients, and this perception was homogeneous between groups (*p* = 0.346). On their last shift, while a similar number of newly admitted/cared for (*p* = 0.511) and discharged patients were cared for (*p* = 0.078), more deaths were encountered in COVID-19 units (average 0.74; CI 95% 0.46–1.06) than in non-COVID-19 units (average 0.14; CI 95% 0.01–0.28) (*p* = 0.021).

**Table 3:** First working experience after graduation (*n* = 323)

Variable	Overall (*n* = 323)	Working in COVID-19 units (*n* = 176)	Working in non-COVID-19 units (*n* = 74)	Unemployed (*n* = 73)	COVID-19 vs. non COVID-19 units *p-value*
Transition programme					
Yes, I have been trained, n (%)	213 (85.2)	145 (82.4)	68 (91.9)	-	0.053
Number of days/shifts, mean (CI95%)	18.7 (15.3- 22.1)	18.4 (14.0–22.7)	19.4 (14.2–24.6)	-	0.783
Reasons for not being trained, n (%)					
Staffing shortages	30 (81.1)	24 (77.4)	6 (100)	-	0.201
Other (e.g., unscheduled training)	7 (18.9)	7 (22.6)	-		
Perceived readiness to be responsible of these patients					
Not at All	10 (4.0)	9 (5.1)	1 (1.3)	-	0.346
Very Little	67 (26.8)	46 (26.1)	21 (28.4)	-	
Somewhat	142 (56.8)	102 (58.0)	40 (54.1)	-	
To a Great Extent	31 (12.4)	19 (10.8)	12 (16.2)	-	
Patients cared for, mean (CI95%)					
Patient’s discharged in the last shift	1.32 (0.92–1.72)	1.24 (0.87–1.60)	1.53 (0.46–2.60)	-	0.511
Patients died in the last shift	0.57 (0.34–0.81)	0.74 (0.42–1.06)	0.14 (0.01–0.28)	-	0.021
Patients admitted/cared for	7.62 (5.74–9.50)	8.69 (6.15–11.23)	4.99 (3.26–6.71)	-	0.078

*CI* Confidence Interval, *COVID-19* COronaVIrus Disease

### Perceived competences

Using the NCS (Table 4), higher degrees of competences were reported in the ‘Helping role’ (average 67.95 out of 100; CI 95% 65.01–70.88), while the lowest were reported for ‘Therapeutic Intervention’ (59.22; CI 95% 56.20–62.25). Regarding the frequency of use, findings were higher for the ‘Helping role’ (average 2.11 out of 3; CI 95% 2.05–2.31) and lower for ‘Teaching-coaching’ (1.58; CI 95% 1.49–1.66). No statistical differences emerged across groups except for the frequency of use during education in ‘Managing situations’, where participants working in non-COVID-19 units reported a higher occurrence (1.96; CI 95% 1.86–2.07) compared to those working in COVID-19 units (1.76; CI 95% 1.54–1.98) (*p* = 0.041). However, no differences emerged in post hoc testing (*p* = 0.069).

**Table 4:** Nurse Competence Scale according to working status and location (*n* = 323)

Factors, mean (CI95%)	Overall (*n* = 323)	Working in COVID-19 units (*n* = 176)	Working in non-COVID-19 units (*n* = 74)	Unemployed (*n* = 73)	COVID-19 vs. non COVID-19 units vs. unemployed *p-value*^*a*^	Post-hoc testing *p-value*^a^^**,**^^b^
Helping role^d^	67.95 (65.01–70.88)	65.17 (58.44–71.89)	67.43 (63.58–71.28)	72.01 (65.83–78.19)	0.282	-
Frequency of using competency^e^	2.11 (2.05–2.31)	2.12 (1.97–2.26)	2.09 (2.01–2.18)	2.17 (2.03–2.31)	0.641	-
Teaching – coaching	62.72 (59.74–65.70)	58.47 (51.74–65.21)	62.25 (58.29–66.22)	68.15 (62.07–74.24)	0.092	-
Frequency of using competency	1.58 (1.49–1.66)	1.50 (1.32–1.69)	1.61 (1.51–1.72)	1.56 (1.36–1.76)	0.571	-
Diagnostic functions	63.59 (60.62–66.55)	61.64 (54.96–68.31)	62.82 (58.85–66.79)	67.41 (61.28–73.55)	0.373	-
Frequency of using competency	1.79 (1.70–1.88)	1.71 (1.50–1.93)	1.87 (1.77–1.98)	1.66 (1.44–1.87)	0.113	-
Managing situations	63.48 (60.53–66.43)	62.12 (55.43–68.82)	62.53 (58.61–66.45)	67.15 (61.03–73.27)	0.415	-
Frequency of using competency	1.86 (1.77–1.95)	1.76 (1.54–1.98)	1.96 (1.86–2.07)	1.70 (1.48–1.92)	0.041	0.069^c^
Therapeutic interventions	59.22 (56.20–62.25)	58.85 (52.09–65.61)	57.65 (53.54–61.75)	63.40 (57.33–69.46)	0.325	-
Frequency of using competency	1.73 (1.64–1.82)	1.69 (1.48–1.90)	1.80 (1.69–1.91)	1.59 (1.36–1.81)	0.161	-
Ensuring quality	62.45 (59.31–65.58)	62.40 (55.46–69.35)	61.08 (56.82–65.34)	65.73 (59.49–72.14)	0.502	-
Frequency of using competency	1.79 (1.69–1.78)	1.74 (1.53–1.95)	1.86 (1.75–1.97)	1.65 (1.42–1.87)	0.154	-
Work role	62.85 (59.83–65.87)	61.18 (54.37–68.00)	61.86 (57.87–65.85)	66.82 (60.56–73.29)	0.352	-
Frequency of using competency	1.84 (1.75–1.93)	1.77 (1.56–1.98)	1.93 (1.82–2.03)	1.68 (1.47–1.90)	0.071	-
Overall competence	63.18 (60.27–66.09)	61.41 (54.80–68.01)	62.23 (58.36–66.10)	67.26 (61.20–73.21)	0.322	-
Frequency of using competency	1.79 (1.70–1.87)	1.72 (1.53–1.92)	1.87 (1.77–1.96)	1.66 (1.45–1.87)	0.113	-

*CI* Confidence Interval, *COVID-19* COronaVIrus Disease

^a^ANOVA Test for continuous variables

^b^Post hoc-testing (tests according to the nature of the variables), working in COVID-19 units vs unemployed

^c^Employed vs. unemployed newly graduates

^d^The NCS, at the competence level, is measured by a visual analogue scale, where 0 indicates a very low level and 100 indicates a high level of competence[22]

^e^The frequency of using the competences increased from ‘Very seldom’ (= 1) to ‘Occasionally’ (= 2) and to ‘Very often’ (= 3)[22]

A data comparison (Table 5) revealed that the perception of some competences was significantly lower among the first COVID-19 new graduate generation (‘Helping role’ 67.95; CI 95% 65.01–70.88 vs. 71.47; CI 95% 68.89–73.06, *p* = 0.036), but significant higher in the ‘Therapeutic intervention’ (59.22; CI 95% 56.20–62.25 vs. 54.02; CI 95% 51.74–56.30, *p* = 0.007) and in ‘Ensuring quality’ (62.45; CI 95% 59.31–65.58 vs. 58.08; CI 95% 55.71–60.45, *p* = 0.028). The frequency of use was significantly lower in the first COVID-19 new graduate generation for ‘Helping role’ (2.11 vs. 2.43, *p* < 0.001), ‘Teaching-coaching’ (1.58 vs. 1.95, *p* < 0.001), ‘Diagnostic functions’ (1.79 vs. 2.08, *p* < 0.001), ‘Managing situations’ (1.86 vs. 2.05, *p* < 0.001) and at the overall level (1.79 vs. 1.99, *p* < 0.001), compared to the pre-COVID-19 generation.

**Table 5:** Nurse Competence Scale of new graduated before and after COVID-19 outbreak

Factors, mean (CI95%)	Pre-COVID-19 generation [21] (*n* = 336)	First COVID-19 new graduate generation (*n* = 323)	*p*-value
Helping role^a^	71.47 (68.89–73.06)	67.95 (65.01–70.88)	0.036
Frequency of using competency^b^	2.43 (2.39–2.47)	2.11 (2.05–2.31)	< 0.001
Teaching – coaching	63.30 (61.40–65.21)	62.72 (59.74–65.70)	0.745
Frequency of using competency	1.95 (1.89–2.00)	1.58 (1.49–1.66)	< 0.001
Diagnostic functions	63.23 (61.25–65.21)	63.59 (60.62–66.55)	0.844
Frequency of using competency	2.08 (2.02–2.13)	1.79 (1.70–1.88)	< 0.001
Managing situations	62.38 (60.25–64.51)	63.48 (60.53–66.43)	0.549
Frequency of using competency	2.05 (1.99–2.11)	1.86 (1.77–1.95)	< 0.001
Therapeutic interventions	54.02 (51.74–56.30)	59.22 (56.20–62.25)	0.007
Frequency of using competency	1.79 (1.72–1.85)	1.73 (1.64–1.82)	0.272
Ensuring quality	58.08 (55.71–60.45)	62.45 (59.31–65.58)	0.028
Frequency of using competency	1.85 (1.78–1.92)	1.79 (1.69–1.78)	0.242
Work role	59.97 (57.81–62.13)	62.85 (59.83–65.87)	0.124
Frequency of using competency	1.83 (1.77–1.88)	1.84 (1.75–1.93)	0.830
Overall competence	61.78 (59.96–63.59)	63.18 (60.27–66.09)	0.419
Frequency of using competency	1.99 (1.95–2.04)	1.79 (1.70–1.87)	< 0.001

*CI* Confidence Interval, *COVID-19* COronaVIrus Disease

^a^The NCS, at the competency level, is measured by a visual analogue scale, where 0 indicates a very low level and 100 indicates a high level of competency [22]

^b^The frequency of using the competences increased from ‘Very seldom’ (= 1) to ‘Occasionally’ (= 2) and to ‘Very often’ (= 3) [22]

## Discussion

To our best knowledge, this is the first study involving the new graduate generation one month after graduation to explore their individual profile, learning experience in the nursing programme during the COVID-19 pandemic and their perceived competences according to employment status, defined as employed in a COVID-19 unit, in a non-COVID-19 unit or unemployed. In other countries, nursing students have been offered extended work in hospitals to support the vast increase in critically ill patients [26, 27]. In Italy, although nursing students have been allowed to attend clinical placements, these have been either suspended for many months or re-initiated with one or two longer placements in some units, albeit with limited hours. In addition, online learning has been offered to compensate for the lack of real-word opportunities, thus transforming the clinical learning strategies offered in the final years of education [23]. All of the unprecedent changes introduced in the nursing programme to face the consequences of the COVID-19 pandemic may have affected new graduates’ perceptions of their own competence as well as their employment status and location one month after graduation.

The profile of the first COVID-19 new graduate generation is similar to that documented recently at both at the national and international levels [21], suggesting that the sample, despite the limited response rate, might reflect the profile of Italian new graduated nurses. Most participants were immediately employed in units caring for COVID-19 patients, with around a quarter employed in non-COVID-19 units and another quarter still unemployed at the time of the survey. Graduates might have perceived a moral obligation to help the system in facing the pandemic [28], also agreeing to work in units caring for COVID-19 patients. Moreover, the majority refused one or more work offers, suggesting that they were given several opportunities and decided to work in COVID-19 or non-COVID-19 units, or to remain unemployed. Interestingly, the reported reasons for refusal included unsatisfactory working conditions/positions, in addition to the distance from home—cited significantly more often among those unemployed as compared to employed. This may suggest the need to stay closer to home—a habit that can be triggered by the sense of insecurity and movement restrictions imposed in the last year to deal with the pandemic [29].

During their last year of education, after the onset of the outbreak, students attended nine weeks of clinical practice on average, with a total of around 300 h (36 h/week). Moreover, around five weeks were spent on distance learning, totalling 180 h (36 h/week). Therefore, a strong reduction in the duration of clinical education was seen, given that > 550 h were used to be required during the third year. Moreover, only a few students have been allowed to learn in COVID-19 units, and the majority were also restricted from accessing these areas, suggesting that they were protected at the cost of learning in a surreal world. Despite their lack of preparation in this field, the majority of new graduates were recruited to COVID-19 units, and these graduates perceived themselves, in some areas of the NCS, even more competent than the other groups did. On the one hand, they may have accepted to work in these units due to a sense of feeling valued by the staff for their contributions, given the uncertain and stressful period in which this study took place. On the other hand, the immediate recruitment into COVID-19 units suggests some reflections. First, there is a need to discuss the ethical implications regarding the restrictions imposed during education and the immediate recruitment just after graduation, as exposing students during their clinical education to a safe learning environment and practice [26] would have allowed them to develop important competences. Second, there is a need to reflect on the quality-of-care received by COVID-19 patients, given the substantial absence of specific clinical experience in caring for these complex patients by new graduates. Third, reflections should be conducted also regarding the safety implications, both at the nursing and system levels, given the significantly lower proportion of COVID-19 units offering a transition programme compared to non-COVID-19 units, mainly due to a shortage of nurses. The recruitment of new graduated nurses into COVID-19 units, which was forbidden during their education, is a point of debate in the attempt to balance clinical placements, including conditions of high risk, and in recruiting nurses to high-risk situations without any previous education or support during their entry-level transition.

The findings from this study indicate that new graduates felt safe in their clinical practice after the onset of COVID-19 in line with the sparse available literature [30], suggesting that they were not exposed to intense stress that might prevent effective learning in line with the sparse literature available [31]. These perceptions did not influence their employment status after graduation. Moreover, most of them were supervised by nurses as previously reported [32], regardless of employment status and location: specifically, how clinical supervision was implemented in times of social distancing merits further investigation. However, during their clinical rotations, few students reported interruptions due to COVID-19 issues (quarantine, isolation), and this was reported more often among those working as new graduates in COVID-19 units. This finding seems to suggest that having personal experience with COVID-19 might have increased their confidence around this clinical issue and thus, their acceptance of working in these challenging settings. By contrast, and surprisingly, participants reporting greater satisfaction with their nursing programmes regarding how the COVID-19 pandemic was managed were significantly more often among those unemployed as compared to those employed, an aspect that should be investigated further.

New graduates reported that they considered themselves ready to undertake the responsibility of nursing care both in COVID-19 and in non-COVID-19 units. Those working in COVID-19 units reported higher exposure to patient death, indicating that specific support when facing stressful situations should be offered alongside the transition programme. Moreover, given that these students have been exposed to a dramatic situation never seen before that may have prepared them to deal with challenging situations, with the return to normality, learnings from this novel situation should be explored to adapt educational programmes and build future preparedness by considering the possible role of simulation and that of disaster preparedness courses [33].

The competences perceived were consistent across groups, suggesting that these did not play a role in the decision of whether to accept a work placement or not, or to work in a COVID-19 unit or not. Findings ranged from > 50 to 75 in the possible range of 0–100, thus reflecting the perception of possessing ‘Good competences’. Only in the ‘Managing situation’ competence new graduates working in COVID-19-units reported a higher frequency of use compared to their colleagues, but this difference (COVID-19 units 1.96; vs. non-COVID-19 units 1.76; unemployed 1.70) does not appear to have a practical relevance.

By contrast, comparing the perceptions of the first COVID-19 new graduate generation with those who graduated previously [21], despite a significantly lower perception of competences having been reported in the ‘Helping role’, others were higher (‘Therapeutic intervention’, ‘Ensuring quality’) or consistent, suggesting that the strategies adopted to ensure the achievement of competences during the pandemic demonstrated some sort of effectiveness. Moreover, new graduates perceived the same degree of overall competence as in the previous study [21], despite their less frequent use of such competences. On the one hand, the lack of clinical opportunities might have maximised the motivation to learn and achieve the expected competences, optimising the learning processes. On the other hand, the increased duration of the clinical placement offered, more than nine weeks in the same unit, might have helped students to develop their competences, to be independent and develop a sense of belonging and closeness to the team—also due to the absence of other students—factors that have all been documented to affect clinical learning outcomes [16, 34]. In this light, the implementation of online education might have supported the initial development of some competences [28], prior to training in the real word, an outcome that should be investigated further.

### Limitations

We involved new graduate nurses with a solid previous clinical education achieved in their first and second years of the programme, which might have compensated for the limited learning opportunities attended in the last year. Moreover, only one formally refused to participate by not giving consent, suggesting that refusals should also be considered among those who did not participate (= 291). The survey was filled in just after graduation, which might have introduced some recall bias regarding the educational opportunities attended in the last year. Furthermore, the survey was stopped after one month; given that it was sent via the official university email, selection bias might have been introduced by including only those graduates still connected to the university. The NCS used [22] was not specific to measuring competences in the middle of a pandemic; therefore, different competences used in daily practice not included in the scale might explain the differences across groups, suggesting that in future surveys, participants should be left free to indicate additional competences they possess. Moreover, competences were self-evaluated and not objectively ranked.

## Conclusion

Since the start of the COVID-19 pandemic, nursing faculties in Italy have undertaken unprecedented decisions to prepare their students to the best level and continue the education of those close to graduation, aiming to respond to the dramatic shortage of the NHS nursing staff. In a context where clinical learning placements have been interrupted by health care facilities, nursing faculties adapted the student educational pathway: one clinical placement, which was longer in duration with no peer education, and preceded by online distance learning, was offered to third year students prior to graduation. At the point of graduation, the majority were employed, mainly in COVID-19 units without a transition programme, imposing an ethical debate regarding the role of nursing education in preparing students for the emerging challenges and the need to ensure the implementation of transition programmes, especially in challenging situations. Alongside an appreciable sign of professionalism from the side of new graduates, nurse managers and executives should consider a moral and legal obligation to protect both new graduates and patients. The competences perceived were homogeneous across employed and unemployed nurses, suggesting that these did not play a role in the decision to accept a work placement or not, or to work in a COVID-19 unit or not. In contrast, comparing the perceived competences of the post-COVID-19 new graduate generation with those of previous graduates, in the same universities, the majority were higher or similar, suggesting that the strategies adopted to ensure the acquisition of competences during the pandemic demonstrated some degree of effectiveness.

Above all, when there is a shortage of something, there is a greater emotional investment and a greater appreciation for what we can have. In the Italian educational tradition, internships have always been experienced as ‘obvious’, which may have reduced students' perceptions of ‘conquering’ and appreciating something as valuable as clinical learning. The pandemic emergency may have helped to highlight the essential nature and importance of clinical learning, where every hour spent in the field can and should generate learning. Future studies in times of the ongoing COVID-19 pandemic could also contribute to the building of a transition programme with a competence scale specific to working in endemics and pandemics to assess and promote preparedness among newly graduated nurses.

## Supplementary Information

**Additional file 1: Supplementary Table 1.** Questionnaire sections and variables. **Supplementary Table 2.** Summary of the data collected among the pre-COVID-19 newly graduates (Kajander-Unkuri et al., 2020).

## Data Availability

According to the quality improvement project intent, at the time of the decision, the local institutional boards recommended to store the database at the institutional level. Therefore, the dataset used and analysed is available from the corresponding author on request.

## Abbreviations

NHS: National Health Service
NCS: Nurse Competence Scale
CI: Confidence Interval
