# Supplementary material for: The first COVID-19 new graduate nurses generation: findings from an Italian cross-sectional study
Source: BMC Nurs. 2022 May 3;21:101. doi: 10.1186/s12912-022-00885-3 (PMC9062856; doi:10.1186/s12912-022-00885-3)
Supplement: Supplementary file 1 — Additional file 1: Supplementary Table 1. Questionnaire sections and variables. Supplementary Table 2. Summary of the data collected among the pre-COVID-19 newly graduates (Kajander-Unkuri et al., 2020). [file 12912_2022_885_MOESM1_ESM.docx]

**Supplementary Table 1**

Questionnaire sections and variables

| Sections   1. Individual variables:  - age, gender, living with, secondary school attended, previous university and work experience(s), academic activities followed at the COVID-19 outbreak, clinical placements attended up to the COVID-19 outbreak onset.  1. Working status one month after graduation:  - working in non-COVID-19 units, working in COVID-19 units, or unemployed.  1. Nursing programme experience from COVID-19 pandemic onset to graduation:  - clinical learning experience attended in the units (weeks), - distance learning experiences attended (weeks), - settings that nursing students were allowed to attend or not (e.g. with COVID-19 patients), - preceptorship model (who supervised the student), - perceived preparedness to deal with the clinical practice and perceived safety while attending clinical settings (4-point Likert scale, from ‘Not at all’ to ‘A great extent’), - interruptions due to quarantine because of contact with COVID-19 patients, clinical teachers or at home (e.g., own family), - isolation due to COVID-19 positivity, - perceived satisfaction with the Nursing Programme regarding the outbreak management (4-point Likert scale, from ‘Not at all’ to ‘A great extent’), - final grade obtained at graduation: from 60 (minimum) to 110 (maximum) cum laude.  1. First working experience as newly graduate:  - whether the transition programme was received or not (e.g. supervised or not by an expert nurse), - for those who have received the transition programme, its duration (in number of shifts), - number of patients admitted/cared for, discharged, and died in the last shift (only for those who were employed), - perceived readiness to undertake the responsibility of patients cared for (4-point Likert scale, from ‘Not at all’ to ‘A great extent’).  1. Perceived competences, as measured with the Nurse Competence Scale (NCS) documented to be capable of measuring the generic competences of nurses as the functional adequacy and capacity to integrate knowledge, skills, attitudes and values in specific contextual situations (Meretoja et al., 2004):  - First section of the NCS tool: It is composed of 73 items for which the nurse is asked to rate the level of competence perceived by using a visual analogue scale (VAS 0‒100; 0=low level, 100=high level of competence). The NCS has been validated in different languages (e.g. Müller, 2013; Wangensteen et al., 2015), including Italian (Dellai et al., 2009; Finotto and Cantarelli, 2009) and it has demonstrated good psychometric properties (Flinkman et al., 2017) also among nursing students (Notarnicola et al., 2018). At the structural validation, the 73 items have been categorised into the following factors: (a) Helping roles (7 items), (b) Teaching – coaching (16 items), (c) Diagnostic functions (7 items), (d) Managing situations (8 items), (e) Therapeutic interventions (10 items), (f) Ensuring quality (6 items) and (g) Working roles (19 items). The average of each factor expresses the overall degree of competences perceived. According to the available evidence (Flinkman et al., 2017; Meretoja et al., 2004), VAS scores <25 indicate ‘Low competence’, >25-50 ‘Rather good competence’, >50-75 ‘Good competence’, and >75-100 ‘Very good competence’. - Second section of the NCS tool: newly graduated nurses were also asked to rank the frequency of use of each competence in their clinical training as 0 = ‘not applicable in my training’; 1 = ‘very seldom used’; 2 = ‘used occasionally’; and 3 = ‘used very often in my work’ (Flinkman et al., 2017, Meretoja et al., 2004). For the context of this study, the option ‘not-applicable’, was not considered. |
| --- |

COVID-19, COronaVIrus Disease; NCS, Nurse Competence Scale; VAS, Visual Analogue Scale.

**References**

Dellai M, Mortari L, Meretoja R. Self‐assessment of nursing competencies–validation of the Finnish NCS instrument with Italian nurses. Scand J Caring Sci. 2009;23(4):783-791. https://doi.org/10.1111/j.1471-6712.2008.00665.x.

Finotto S, Cantarelli W. Nurse's competence indicators: linguistic and cultural validation of the Nurse Competence Scale. Prof Inferm. 2009;62(1):41-48.

Flinkman M, Leino-Kilpi H, Numminen O, Jeon Y, Kuokkanen L, Meretoja R. Nurse Competence Scale: a systematic and psychometric review. J Adv Nurs. 2017;73 (5): 1035-1050. http://dx.doi.org/10.1111/jan.13183.

Meretoja R, Isoaho H, Leino-Kilpi H. Nurse competence scale: development and psychometric testing. J Adv Nurs. 2004;47(2):124-133. http://dx.doi.org/10.1111/j.1365-2648.2004.03071.x.

Müller M. Nursing competence: psychometric evaluation using Rasch modelling. J Adv Nurs. 2013;69(6): 1410-1417. http://dx.doi.org/10.1111/jan.12009.

Notarnicola I, Stievano A, De Jesus Barbarosa MR, Gambalunga F, Iacorossi L, Petrucci C, Pulimeno A, Rocco G, Lancia L. Nurse Competence Scale: psychometric assessment in the Italian context. Ann Ig. 2018;30(6):458-469. http://dx.doi.org/10.7416/ai.2018.2246.

Wangensteen S, Johansson IS, Nordstrom G. Nurse Competence Scale--psychometric testing in a Norwegian context. Nurse Educ Pract. 2015;15(1):22-29. http://dx.doi.org/10.1016/j.nepr.2014.11.007.

**Supplementary Table 2**

Summary of the data collected among the pre-COVID-19 newly graduates (Kajander-Unkuri et al., 2020)

| Study design  Data collection  Sample  Participants  Time of data collection  Nurse Competence Scale  Questionnaire  Pilot  Methods of data collection  Main results (Italy) | A cross-sectional evaluative design  From February 2018 to July 2019 (in Italy from October 2018 to March 2019)  Convenience sampling  335 out of 345 Italian nursing students (response rate = 97.1%)  At the time of graduation (Italy)  The Nurse Competence Scale (Meretoja et al., 2004) was validated in a preliminary fashion  Background factors (age, gender, previous work and university experiences)  Performed in each country involved in the study  Online survey, after having informed new graduates regarding the study aims and procedures  - Age (years), mean (SD) = 23.6 (3.2)  - Gender (female), n. (%) = 274 (81.8)  - Work experience in health care besides clinical practice during nursing education (yes), n. (%) = 113 (33.7)  - Satisfaction with current nursing education programme as whole (satisfied/very satisfied), n. (%) = 313 (93.4) |
| --- | --- |

COVID-19, COronaVIrus Disease; SD, Standard Deviation.

**References**

Kajander-Unkuri S, Koskinen S, Brugnolli A, Torre MC, Elonen I, Kiele V, Lehwaldt D, Loyttyniemi E, Nemcova J, de Oliveira CS et al. The level of competence of graduating nursing students in 10 European countries-Comparison between countries. Nurs Open. 2021 May;8(3):1048-1062.
